# Effects of Telerehabilitation on Patient Adherence to a Rehabilitation Plan: Protocol for a Mixed Methods Trial

**DOI:** 10.2196/32134

**Published:** 2021-10-28

**Authors:** Isabelle Gaboury, Michel Tousignant, Hélène Corriveau, Matthew Menear, Guylaine Le Dorze, Christian Rochefort, Brigitte Vachon, Annie Rochette, Sylvie Gosselin, François Michaud, Jessica Bollen, Sarah Dean

**Affiliations:** 1 Department of Family Medicine and Emergency Medicine Faculty of Medicine and Health Sciences Université de Sherbrooke Longueuil, QC Canada; 2 School of Rehabilitation Faculty of Medicine and Health Sciences Université de Sherbrooke Sherbrooke, QC Canada; 3 Department of Family Medicine and Emergency Medicine Faculty of Medicine Université Laval Québec, QC Canada; 4 School of Audiology and Speech Therapy Faculty of Medicine Université de Montréal Montréal, QC Canada; 5 School of Nursing Faculty of Medicine Université de Sherbrooke Sherbrooke, QC Canada; 6 School of Rehabilitation Faculty of Medicine Université de Montréal Montreal, QC Canada; 7 Centre for Interdisciplinary Research in Rehabilitation of Greater Montreal (CRIR) Montreal, QC Canada; 8 Department of Medicine Faculty of Medicine and Health Sciences Université de Sherbrooke Sherbrooke, QC Canada; 9 Department of Electrical and Computer Engineering Faculty of Engineering Université de Sherbrooke Sherbrooke, QC Canada; 10 Institute of Health Research University of Exeter Medical School Exeter United Kingdom

**Keywords:** adherence, interprofessional shared decision making, rehabilitation, stroke, telerehabilitation

## Abstract

**Background:**

Strong evidence supports beginning stroke rehabilitation as soon as the patient’s medical status has stabilized and continuing following discharge from acute care. However, adherence to rehabilitation treatments over the rehabilitation phase has been shown to be suboptimal.

**Objective:**

The aim of this study is to assess the impact of a telerehabilitation platform on stroke patients’ adherence to a rehabilitation plan and on their level of reintegration into normal social activities, in comparison with usual care. The primary outcome is patient adherence to stroke rehabilitation (up to 12 weeks), which is hypothesized to influence reintegration into normal living. Secondary outcomes for patients include functional recovery and independence, depression, adverse events related to telerehabilitation, use of services (up to 6 months), perception of interprofessional shared decision making, and quality of services received. Interprofessional collaboration as well as quality of interprofessional shared decision making will be measured with clinicians.

**Methods:**

In this interrupted time series with a convergent qualitative component, rehabilitation teams will be trained to develop rehabilitation treatment plans that engage the patient and family, while taking advantage of a telerehabilitation platform to deliver the treatment. The intervention will be comprised of 220 patients who will take part in stroke telerehabilitation with an interdisciplinary group of clinicians (telerehabilitation group) versus face-to-face standard of care (control group: n=110 patients).

**Results:**

Our Research Ethics Board approved the study in June 2020. Data collection for the control group is underway, with another year planned before we begin the intervention phase.

**Conclusions:**

This study will contribute to the minimization of both knowledge and practice gaps, while producing robust, in-depth data on the factors related to the effectiveness of telerehabilitation in a stroke rehabilitation continuum. Findings will inform best practice guidelines regarding telecare services and the provision of telerehabilitation, including recommendations for effective interdisciplinary collaboration regarding stroke rehabilitation.

**Trial Registration:**

ClinicalTrials.gov NCT04440215; https://clinicaltrials.gov/ct2/show/NCT04440215

**International Registered Report Identifier (IRRID):**

DERR1-10.2196/32134

## Introduction

Stroke impacts nearly 400,000 Canadians annually [1]. It is the leading cause of adulthood disability and is associated with substantial morbidity and mortality [2]. Three-quarters of stroke survivors will live with minor to severe impairments or disabilities, requiring rehabilitation services [3]. Strong evidence supports beginning rehabilitation as soon as the patient’s status has stabilized and continuing following discharge from acute care [4]. Returning home shortly after a stroke event places the patient in the most favorable environment to foster the success of the rehabilitation therapy [5] and should be favored over inpatient rehabilitation [6]. For this reason, enabling access and optimizing adherence to rehabilitation services is crucial to ensure positive patient and family outcomes [4]. That said, the need to travel long distances regularly to attend rehabilitation sessions with various professionals [7], the lack of coordination and communication among these outpatient services [4], and the failure to engage the patient and family members in a structured decision-making process [8] limit uptake and delivery of optimal services.

Telerehabilitation, which refers to the use of technology to provide long-distance rehabilitative services [9], is recommended by the Canadian Stroke Guidelines as a means to ensure equal and timely access to optimal stroke services [6]. In this research project, we focus on teletreatment, the provision of remote interprofessional rehabilitation and communication services using videoconferencing technologies.

Despite emerging evidence on the clinical and economic benefits of telerehabilitation [10,11], knowledge gaps remain, especially in a population of community-dwelling stroke patients [12]. Promising results following telerehabilitation include improved function [13-15] and recovery from motor deficits, social function [16,17], quality of life [18], and depression scores [16]. These studies, however, are often characterized by very small sample sizes (ie, fewer than 50 patients investigated), suboptimal treatment length (eg, 1-month duration, whereas guidelines recommend 8-12 weeks), or various definitions of what telerehabilitation entails [10,15,19-27]. As a result, many benefits of the intervention still need to be investigated using robust and large trials [12,14,28-32].

Furthermore, interprofessional shared decision making (SDM) has been shown to help teams to deliver better-quality care and resulted in significant improvements in patients’ rehabilitation process [33,34] (eg, patients’ knowledge and understanding, participation in the decision-making process, and satisfaction with and trust in the health care team). SDM refers to the process by which health decisions are deliberated upon and made jointly by the patient and one or more health professionals, taking into consideration the best available evidence and the patient’s values and preferences [35,36]. Although the effects of SDM have not been extensively documented [33,37] in stroke care, shared decisions are considered the crux of patient-centered care, and SDM has been correlated with greater adherence to treatment plans in other populations [38-43]. Structured interactions among team members through a telerehabilitation platform can create a unique opportunity to improve interprofessional communication and diagnosis skills by supporting the simultaneous participation of all team members, including the patients and family [43,44].

We propose a mixed methods clinical trial to assess the effectiveness of a telerehabilitation platform to increase adherence to rehabilitation stroke care as well as to increase patient participation in interprofessional SDM. The specific objectives of this project are to (1) evaluate the clinical, process, and cost outcomes of an interprofessional technology-enabled stroke rehabilitation intervention in comparison with usual care and (2) identify and describe key contextual factors related to the outcomes of this intervention.

## Methods

### Study Design

An interrupted time series design with a convergent qualitative component is proposed to test the effectiveness of the intervention. The intervention consists of observing the same dependent variable over time, with a break in the series of observations corresponding to the introduction of an intervention. If the intervention is effective, a change in the series’ pre- and postintervention averages can be observed [45]. Moreover, such design will accommodate pre-existing trends and control for possible variations within and among sites (eg, rehabilitation treatment efficacy, low adherence to treatment plan, and team composition). This study was registered at ClinicalTrials.gov (NCT04440215).

### Setting

Five selected sites in Quebec, Canada, will start with a control period (preintervention: usual care) until they independently reach their target sample size; this is expected to take between 12 and 18 months. Participants recruited during this period will belong to the control group. The sites will then enter the intervention period until recruitment completion, which is expected to occur after a further 12 to 18 months. Participants recruited during this second period will belong to the intervention group. Site monitoring will carefully document practices during both trial phases.

### Participants

The study population consists of male and female adults, 18 years and older, who (1) have had a stroke event, (2) are considered to be safe for home discharge by the acute or inpatient care team, (3) have a relative or caregiver who is present in the home should physical rehabilitation treatments be required, and (4) can speak French or English. Patients with severe cognitive decline prior to the stroke event will be excluded. Patients with communication difficulties resulting from the stroke event (eg, aphasia) will not be excluded. When possible, a patient’s relative or informal caregiver will also be recruited to document their experience of care. Inclusion and exclusion criteria will be similar to above with the exception of the stroke event [46].

Patients will be recruited at each rehabilitation center. At admission, the rehabilitation care coordinator will identify eligible patients and will present the study to the patients and their families. The consent form will be signed prior to the first intervention session. A research assistant will contact the patient and family by telephone to confirm consent and for data collection.

### Intervention

#### Control Phase

Rehabilitation teams will be instructed to provide care as they have been doing previously (ie, usual care). This translates into (1) no telerehabilitation, (2) interdisciplinary meetings not systematically organized and/or not involving a complete team of professionals, and (3) care plans not necessarily elaborated using interprofessional SDM principles.

#### Intervention Phase

The telerehabilitation platform will be installed at each site in rooms devoted to telerehabilitation activities and equipped with rehabilitation gears. The software OpenTera is a cloud-based multipoint, multi-view, and multi-stream (ie, video and audio) telecommunication system with proven usability and robustness for telerehabilitation applications [7]. The platform is not linked to a specific commercial platform or to special network configurations. It supports multisite interventions (ie, more than one patient at a time), making group sessions and interprofessional meetings feasible. A second camera will be installed on both clinician and patient kits for patients needing speech therapy. The rehabilitation team members will be trained to adapt rehabilitation exercises. Preintervention and ongoing training and coaching will be provided by a trainer who has extensive experience of training rehabilitation teams for various health care conditions; this will be done using the platform.

All rehabilitation teams will receive training in an interprofessional approach to SDM, which recognizes that multiple professionals may be involved in care planning and can support patients and their families in making decisions that are right for them. The teams will receive training to promote knowledge and skills on these topics, and sessions will be modeled after the interprofessional training program used successfully in two recent trials of SDM among home care teams [47,48]. The 3-hour program has two components: (1) a general online tutorial on SDM, based on the Ottawa Decision Support Tutorial [49], and (2) a skill-building workshop that includes a lecture, a video, and a role-play exercise. The brief lecture will be delivered by two experts in SDM and will provide definitions and a conceptual framework for the interprofessional SDM approach. The video will present a clinical vignette illustrating how the interprofessional SDM approach translates into a clinical scenario involving a patient and multiple professionals working together to collectively support a decision. The role-playing exercise will then allow participants in the training session to work in small groups and put into practice lessons learned using a fictional scenario. For this study, materials for the lecture and role-play exercise will be adapted and tailored to the stroke care context, including best practices in care planning as well as SDM [50].

From the patient’s perspective, a mix of home or rehabilitation center visits and telerehabilitation will be planned by the rehabilitation team for a maximum of 16 weeks. The rehabilitation services offered will be based on availability at each site. Evaluation, re-evaluation, and manual therapy treatments will be done face-to-face. To ensure internal validity, telerehabilitation sessions are expected to represent at least 80% of participants’ rehabilitation plans.

For each participant enrolled, at least one multidisciplinary meeting will be organized to present the rehabilitation treatment plan. The patient and family will participate in the meeting and the decision-making process using the telerehabilitation platform. The team will generate an interprofessional individualized treatment plan for each enrolled participant. A randomized sample of 60 meetings—30 from the control group and 30 from the intervention group—will be selected for recording. This type of nonparticipant observation will allow for a better understanding of the process of interprofessional SDM over the course of the trial.

### Primary Outcome

The choice of the primary outcome, patient adherence to the stroke rehabilitation plan at 12 weeks, was identified through a pilot study previously conducted by our research team as the most meaningful outcome and to better document the reason why telerehabilitation might be effective. Adherence to telerehabilitation has been operationalized in many different ways across studies [6,51]. We will define adherence as time spent (in minutes) doing any stroke rehabilitation exercises (online + offline). This includes, but is not limited to, physical, writing, and speech therapy as well as mental health–related exercises recommended by the rehabilitation professional. Online session time will be recorded through the telerehabilitation platform. Offline time will include face-to-face sessions doing rehabilitation as well as time exercising on one’s own as instructed by the rehabilitation professional. Offline sessions completed at home will be captured with the use of a journal and recorded by one of the rehabilitation professionals each week. This monitoring step has been used as a method of quality assurance by other scholars in stroke research [52]. Patients’ adherence to a rehabilitation program will also be measured by the Stroke Rehabilitation Exercise Adherence Measure (StREAM) questionnaire at weeks 4, 6 (to allow for test-retest reliability assessment), and 12 [53]. Moreover, health care professional perception of the participants’ adherence to the rehabilitation program will be evaluated on a numerical 10-point rating scale each week during the intervention period.

### Secondary Outcomes

Secondary clinical outcomes, the instruments used to measure them, and times of measurement are listed in Table 1 [46,54-63].

Focus groups will be conducted with 6 to 12 purposefully selected rehabilitation team members from each site at the end of the intervention phase. Stroke rehabilitation team members will be selected to capture a variety of professions and roles within the team and to ensure information-rich discussions. Themes such as the attributes of telerehabilitation, facilitating contextual conditions, opinion leadership, and platform adaptation for both clinical and collaborative work will be explored with patients and families as well as professionals. Quarterly in-depth interviews with the five site clinical champions will document their ongoing experience.

Similarly, qualitative interviews with 5 to 8 purposefully selected intervention patients and their families, when available, per site will further document the patients’ and families’ experiences of the technology, interprofessional SDM, and relationship with their outcomes.

**Table 1:** Secondary outcomes.

Outcome		Instrument	Time of measurement
**Patients**			
	Reintegration into normal life	Reintegration to Normal Living Index [54]	Baseline and 12 weeks
	Ability to perform daily activities	Functional Independence Measure [46]	Baseline and 12 weeks
	Participation in shared decision making (SDM)	Questionnaire by Strull et al [55]	Following the establishment of the patient treatment plan
	Decisional conflict	SURE (Sure of myself; Understand information; Risk-benefit ratio; Encouragement) questionnaire [56]	Following the establishment of the patient treatment plan
	Satisfaction with treatment plan	Satisfaction with Decision scale [57]	Following the establishment of the patient treatment plan
	Depression	The Beck Depression Inventory [58]	Baseline, 12 weeks, and 24 weeks
	Satisfaction with health care received	Health Care Satisfaction Questionnaire [59]	12 weeks
**Health care use**			
		Institut national de santé publique du Québec population questionnaire (section A) [60]	12 and 24 weeks
		Provincial health administrative data	12 and 24 weeks
	Adverse events	Patient’s calendar (collected weekly by a rehabilitation professional): incidence of falls, dizziness, pain (visual analog scale), and fatigue (Borg Rating of Perceived Exertion Scale, 1-10)	Weekly basis up to 12 weeks
	Individual costs	Questionnaire developed by the authors for this study	24 weeks
**Patient’s relatives**			
	Quality of services received	Quality of Services Questionnaire for Relatives poststroke [61]	12 weeks
**Rehabilitation professionals**			
	Statistics related to the use of the telerehabilitation platform (who, when, and duration)	Questionnaire developed by the authors for this study	Weekly basis
	Interprofessional collaboration	Assessment of Interprofessional Team Collaboration Scale, short version [62]	Every 3 months for the full duration of the study
	Perception of interprofessional care SDM	Collaboration and Satisfaction About Care Decisions questionnaire [63]	Following the establishment of the patient treatment plan

### Sample Size Estimates

We calculated the sample size for a univariate comparison of the primary outcome between the two study groups at 12 weeks. The required sample size was calculated using G*Power (version 3.1.9.4; Heinrich-Heine-Universität Düsseldorf) based on a conservative estimated effect size of the intervention (Cohen *d*) of 0.4. This sample size was calculated assuming the following: (1) a two-sided type I error probability of 5% and a power of 80%, (2) a duration of 12 weeks, (3) a 2:1 ratio of intervention to control group participants, (4) two study groups, (5) a <5% loss to follow-up, and (6) intracluster correlations of 0.10. Therefore, 330 participants will be recruited: 110 and 220 participants in the control and intervention groups, respectively.

### Analyses

Baseline site and participant characteristics will be summarized descriptively. Patient outcomes will be summarized by group and site. Statistical analysis of the data will follow intention-to-treat principles. A linear mixed model will be used, with individuals’ data nested into a study site, to isolate the effect of the intervention on both clinical and process outcomes from other changes that may take place during the trial. Key contextual factors, such as patient’s sex and age, state of employment prior to stroke, stroke severity, concomitant rehabilitation treatments received outside the rehabilitation center, and time since stroke event (in days), will be considered in regression models as potential confounders. Interaction terms will be included in the model for any statistically significant contextual factors. Presence of dichotomous stroke outcomes (eg, dizziness) will be compared between study groups using a chi-square test. Multiple imputation will be considered if missing data represent more than 10% of a given variable. Sensitivity analysis will be conducted to assess the impact of missing data on estimates of intervention effects with a multiple imputation statistical technique and without imputation (ie, available case analyses), as well as for outlying observations.

Interviews and treatment plan meetings will be fully transcribed. A descriptive analytical approach will be used to develop a framework of organizational factors leading to a successful telerehabilitation intervention, both at the patient and professional levels. Audio recordings and transcripts will be reviewed simultaneously to assess validity of the transcription process and will be analyzed using an iterative approach [64]. Data saturation will be determined as defined by Constantinou et al [65]. This will be followed by an intersite analysis to identify what is common between the sites examined and what is specific to certain sites, and to compare the different configurations between sites. Finally, matrices with contextual factors will be generated to identify particular patterns [66].

### Ethics

The Research Ethics Board of the Hôpital Charles-Le Moyne of the CISSS (Centre intégré de santé et des services sociaux) de la Montérégie-Centre has approved this research project and is providing oversight on the ethical concerns of this project, including for any potential revisions of the protocol. Patients and caregivers have both been thoroughly informed of all aspects of the research protocol in which they might be included and have been assigned a patient number for anonymization purposes. All data will be collected by phone or using paper questionnaires. Data will be kept in a password-protected database in the research team’s private servers, and paper questionnaires will be kept in a locked container in the offices of the research team.

## Results

As of July 2021, a total of 37 patients have been enrolled, from which 12 patients have completed the study. Data collection for the control group is expected to last for another 6 to 12 months; this will be completed before we begin the intervention group data collection, which should last 2 to 3 years. We do not intend to begin analysis before the end of the data collection period.

## Discussion

### Impact

From a clinical perspective, the use of a telerehabilitation platform will improve adherence to stroke rehabilitation programs by (1) better anchoring patients and families in their own environments (eg, by using day-to-day objects to perform rehabilitation exercises) to favor functional rehabilitation; (2) facilitating an intensive rehabilitation program by decreasing the time and hurdles of traveling; (3) providing an optimal care plan that matches the patient and family condition, context, and expectations; and (4) favoring more active participation by the patient and family as well as by all members of the rehabilitation team.

### Limitations

Precautions will be taken to train each stroke team immediately prior to the intervention launch, to minimize contamination between the control and the intervention phases, which is one of the main risks of this study. The use of a linear mixed model should prevent an overestimation of the effect of the intervention [67], a common limitation of study designs that take place over a long period of time.

Another potential weakness is that organizational change entails an inner shift in the organization’s stakeholder values and aspirations as well as a series of behavior changes in response to external shifts in processes, strategies, and environments [68]. The proposed intervention will require adjustments from the rehabilitation professionals in order to gradually shift the teams’ ways of delivering care. Frequent scheduled interactions between rehabilitation and study teams should create an empowering context for the care providers involved in this trial; minimize anxiety, resistance, and unproductive behaviors; and ensure that the intervention implementation is rooted in the organization’s culture.

Findings from this study will inform best practices guidelines by providing empirical data on effective collaboration processes as well as optimized telerehabilitation delivery via telecare services. The production of a guideline called “How to better implement telerehabilitation within a stroke continuum” with dissemination through governmental agencies will aid sites not involved in this trial to implement telerehabilitation, in addition to classical dissemination, such as conferences and journal publications. This will also optimize the impacts of this intervention on stroke rehabilitation continuums beyond the trial sites.

This study represents a unique, highly relevant, and innovative opportunity to minimize both knowledge and practice gaps in rehabilitation stroke care, including interprofessional SDM. This study will produce robust data on the effectiveness of the intervention and in-depth data on the contextual factors and mechanisms that are related to its effectiveness, for whom and how. Participating health care providers will gain the wherewithal to engage patients and families and to develop their interprofessional decision-making skills, which are crucial to meet patients’ needs and significantly improve patient adherence.

## Appendix

Multimedia Appendix 1 Peer-review report by the Canadian Institutes of Health Research/Instituts de recherche en santé du Canada (CIHR/IRSC).

## Abbreviations

CISSS: Centre intégré de santé et des services sociaux
SDM: shared decision making
StREAM: Stroke Rehabilitation Exercise Adherence Measure
