# Supplementary material for: Effects of Telerehabilitation on Patient Adherence to a Rehabilitation Plan: Protocol for a Mixed Methods Trial
Source: JMIR Res Protoc. 2021 Oct 28;10(10):e32134. doi: 10.2196/32134 (PMC8587325; doi:10.2196/32134)
Supplement: Multimedia Appendix 1 [file resprot_v10i10e32134_app1.pdf]

January/Janvier 22, 2020

DC0190GP

Canadian Institutes of Health Research / Instituts de recherche en santé du Canada

Notice of Decision / Avis de décision

Application Number/Numéro de la demande: 425748

Committee Code/Code du comité: MOV

Applicants/Candidats: Professeure Isabelle Gaboury | Monsieur Sylvain Gobeil | Professeur Michel Y. Tousignant

With/Avec: Professeure H. Corriveau | Docteure S. Gosselin | Docteure G. Le Dorze | Dr. M. Menear | Professeur F. Michaud | Dr. C. Rochefort | Madame A. Rochette | Madame B. Vachon

Institution paid/ Établissement payé: Université de Sherbrooke

Title/Titre: Optimizing patient adherence to stroke rehabilitation treatment: a telerehabilitation trial

Primary Inst./ Inst. principal: Circulatory and Respiratory Health / Santé circulatoire et respiratoire

Other Related Inst./ Autres inst. connexes: Health Services and Policy Research / Services et politiques de la santé; Aging / Vieillissement

Competition Outcome/Résultats du concours: Project Grant / Subvention Projet

September/Septembre 11, 2019

Number in competition/Nbre de demandes dans le concours: 2183

Number approved/Nbre de demandes approuvées: 385

Decision on your application/ Décision sur votre demande: Approved / Approuvée

Total Funding Amount:/ Montant total du financement: $696,150

Term/Durée: **5 yrs/ans 0 months/mois**

Peer Review Committee Recommendation, for your information and use/ Recommandation du comité d'examen par les pairs, pour fins d'information et d'utilisation:

Committee/Comité: Movement & Exercise / Mouvement et exercice

Number reviewed/ Nbre de demandes examinées: 37

Number approved in that committee/ Nbre de demandes approuvées dans ce comité: 6

Application rank within the committee/ Rang de la demande dans ce comité: 3

Percent Rank Within the Committee/ Rang en pourcentage au sein du comité: 94.44%

Rating/ Cote: 4.74

Additional Funding Opportunities/ Opportunités de financement additionnelles	Decision/ Décision	Total Funding Amount/Montant total du financement	Competition Code/Cote de concours	Application Number/ Numéro de la demande
Project Grant - Priority Announcement: Health Services and Policy Research/Subvention Projet - AP : Recherche sur les services et les politiques de santé	Non approuvée/ Not Approved	$0	201909PJI	431760
Project Grant - Priority Announcement: Aging/Subvention Projet - Annonce de priorités: Vieillissement	Non approuvée/ Not Approved	$0	201909PJ2	431761

This document is for information only. Official payment is stated on the CIHR Authorization for Funding.
Document à titre d'information seulement. Le paiement officiel est indiqué sur l'autorisation de financement des IRSC.

Canadian Institutes of Health Research
160 Elgin Street, 9th Floor
Address Locator 4809A
Ottawa, Ontario K1A 0W9

Instituts de recherche en santé du Canada
160, rue Elgin, 9e étage
Indice de l'adresse 4809A
Ottawa (Ontario) K1A 0W9

Institute of Aging
Institute of Cancer Research
Institute of Circulatory and Respiratory Health
Institute of Gender and Health
Institute of Genetics
Institute of Health Services and Policy Research
Institute of Human Development and Child and Youth Health
Institute of Indigenous Peoples' Health
Institute of Infection and Immunity
Institute of Musculoskeletal Health and Arthritis
Institute of Neurosciences, Mental Health and Addiction
Institute of Nutrition, Metabolism and Diabetes
Institute of Population and Public Health

Institut du vieillissement
Institut du cancer
Institut de la santé circulatoire et respiratoire
Institut de la santé des femmes et des hommes
Institut de génétique
Institut des services et des politiques de la santé
Institut du développement et de la santé des enfants et des adolescents
Institut de la santé des Autochtones
Institut des maladies infectieuses et immunitaires
Institut de l'appareil locomoteur et de l'arthrite
Institut des neurosciences, de la santé mentale et des toxicomanies
Institut de la nutrition, du métabolisme et du diabète
Institut de la santé publique et des populations

Le 22 janvier 2020

Professeure Isabelle Gaboury
Département de médecine de famille et d'urgence
Université de Sherbrooke
Campus Longueuil
150 Place Charles-LeMoyne
Longueuil, Québec J4K 0A8

Professeure Gaboury,

Au nom des Instituts de recherche en santé du Canada (IRSC), j'ai le plaisir de vous informer que votre demande intitulée « Optimizing patient adherence to stroke rehabilitation treatment: a telerehabilitation trial », présentée au concours de subventions Projet de l'automne 2019, a été approuvée pour du financement.

Les évaluations et les résultats de votre demande sont accessibles dans RechercheNet. Si vous ne pouvez accéder aux documents, veuillez communiquer avec nous à support-soutien@cihr-irsc.gc.ca. Votre autorisation de financement vous sera envoyée par la poste.

Il convient de souligner que le nombre de demandes approuvées (indiquées dans l'avis de décision) comprend les demandes figurant au-dessus du seuil de financement établi pour le comité et, le cas échéant, toute demande de chercheurs en début de carrière (CDC) qui figurait sous le seuil de financement, mais qui a été financée en entier par les fonds spécialement prévus pour le rééquilibrage des taux de réussite des CDC. Ce processus de rééquilibrage permet aux IRSC de s'assurer que les CDC sont financés en proportion égale aux candidats CDC au concours de subventions Projet; autrement dit, les candidats CDC affichent des taux de réussite semblables à ceux des candidats chevronnés ou en milieu de carrière.

Étant donné que les IRSC n'informent pas les cocandidats de leur décision, nous vous prions de communiquer le résultat de cette demande aux personnes concernées et à leurs établissements de recherche (s'ils diffèrent du vôtre).

Pour toute question, n'hésitez pas à communiquer avec un agent de traitement du centre de contact par téléphone au 613-954-1968 ou par courriel à support-soutien@cihr-irsc.gc.ca.

Nous vous félicitons pour votre réussite à ce concours.

Sincères salutations,

Nathalie Gendron

Nathalie Gendron, Ph.D.
Gestionnaire, Conception et exécution des programmes
Portefeuille des programmes de recherche

491113-201909PJT-MOV-425748-106520-DLPJT

Canadian Institutes of Health Research
160 Elgin Street, 9th Floor
Address Locator 4809A
Ottawa, Ontario K1A 0W9

Instituts de recherche en santé du Canada
160, rue Elgin, 9e étage
Indice de l'adresse 4809A
Ottawa (Ontario) K1A 0W9

Institute of Aging
Institute of Cancer Research
Institute of Circulatory and Respiratory Health
Institute of Gender and Health
Institute of Genetics
Institute of Health Services and Policy Research
Institute of Human Development and Child and Youth Health
Institute of Indigenous Peoples' Health
Institute of Infection and Immunity
Institute of Musculoskeletal Health and Arthritis
Institute of Neurosciences, Mental Health and Addiction
Institute of Nutrition, Metabolism and Diabetes
Institute of Population and Public Health

Institut du vieillissement
Institut du cancer
Institut de la santé circulatoire et respiratoire
Institut de la santé des femmes et des hommes
Institut de génétique
Institut des services et des politiques de la santé
Institut du développement et de la santé des enfants et des adolescents
Institut de la santé des Autochtones
Institut des maladies infectieuses et immunitaires
Institut de l'appareil locomoteur et de l'arthrite
Institut des neurosciences, de la santé mentale et des toxicomanies
Institut de la nutrition, du métabolisme et du diabète
Institut de la santé publique et des populations

Le 22 janvier 2020

Professeure Isabelle Gaboury
Département de médecine de famille et d'urgence
Université de Sherbrooke
Campus Longueuil
150 Place Charles-LeMoyne
Longueuil, Québec J4K 0A8

Bonjour,

Au nom des Instituts de recherche en santé du Canada (IRSC), j'aimerais vous féliciter de votre succès au récent concours de financement des IRSC!

En tant que chercheurs du domaine de la santé, nous partageons un objectif commun : améliorer la santé et le bien-être des populations, au Canada et dans le monde. Par l'entremise des IRSC, le gouvernement du Canada offre un soutien essentiel aux chercheurs canadiens œuvrant dans tous les thèmes de la recherche en santé, qui sont étroitement liés, afin d'améliorer la santé de toute la population canadienne. Titulaire d'une subvention des IRSC, vous faites maintenant partie de cette entreprise.

Vous n'êtes pas sans savoir que l'évaluation de votre demande de subvention a été possible grâce aux pairs évaluateurs qui ont généreusement donné de leur temps pour soutenir le secteur canadien de la recherche en santé. À titre de titulaire de fonds des IRSC, vos connaissances et votre expertise sont fort précieuses. C'est pourquoi je vous invite à envisager de devenir membre du Collège des évaluateurs, si vous ne l'êtes pas déjà. Veuillez consulter la page www.cihr-irsc.gc.ca/f/49923.html pour prendre connaissance des critères de sélection et pour savoir comment soumettre votre candidature.

Aujourd'hui, l'activité scientifique est surveillée de près et il est plus important que jamais de veiller à ce que notre travail soit bien compris. Je vous demande donc de faire mention du financement des IRSC dans vos présentations et vos communications au sujet de vos recherches, et de continuer de faire valoir l'importance cruciale de la recherche pour la santé des Canadiennes et des Canadiens.

Encore une fois, je tiens à vous féliciter de cet exploit et à vous offrir mes meilleurs vœux de succès dans toutes vos entreprises. Je suivrai avec intérêt l'avancement de vos recherches.

Je vous prie de recevoir mes plus cordiales salutations.

Michael J. Strong, M.D., FRCPC, FAAN, MACSS
Président

491116-201909PJT-MOV-425748-106520-CONGR

Canada

CIHR IRSC
Discoveries for life / Découvertes pour la vie

**Review Type / Type d'évaluation:**	Reviewer 1 / Évaluateur 1
**Name of Applicant / Nom du chercheur:**	Gaboury, Isabelle
**Application No. / Numéro de demande:**	425748
**Agency / Agence:**	CIHR/IRSC
**Competition / Concours:**	Project Grant/Subvention Projet
**Committee / Comité:**	Movement & Exercise/Mouvement et exercice
**Title / Titre:**	Optimizing patient adherence to stroke rehabilitation treatment: a telerehabilitation trial

Adjudication Criteria/Critères de sélection

Significance and Impact of the Research/Importance et impact de la recherche: 4.6

Approaches and Methods/Approches et méthodes: 4.6

Expertise, Experience and Resources/Expertise, expérience et ressources: 4.8

Top/Bottom Selection/Groupe supérieur/inférieur

- ◉ **Top/Groupe supérieur**
- ○ **Bottom/Groupe inférieur**

**Review Type / Type d'évaluation:**	Reviewer 1 / Évaluateur 1
**Name of Applicant / Nom du chercheur:**	Gaboury, Isabelle
**Application No. / Numéro de demande:**	425748
**Agency / Agence:**	CIHR/IRSC
**Competition / Concours:**	Project Grant/Subvention Projet
**Committee / Comité:**	Movement & Exercise/Mouvement et exercice
**Title / Titre:**	Optimizing patient adherence to stroke rehabilitation treatment: a telerehabilitation trial

Summary of Application/Résumé de la demande:

This is a third re-submission for this proposal. This study is a pragmatic clinic trial using an interrupted time series design. The team aims to enrol 296 patients at a time when they are ready to be discharged from the hospital after a stroke, and their family caregivers. Half of the patients will receive rehabilitation through remote, live treatment sessions with an interdisciplinary team (telerehabilitation). The interdisciplinary team will hold regular meeting and provide a treatment plan that is jointly developed with the patient. The other half will receive the standard care, which has no telerehabilitation, no routine interdisciplinary team meeting, and may not receive an inter-professional care plan (expecting fewer than 2/3 will receive this).

Five rehabilitation sites will participate in this study. They will provide the standard care to approximately 40 patients (recruitment 12 – 18 months). Then they will receive training on the telerehab platform to engage the patient/family, on shared decision making, and on how to develop rehabilitation treatment plans that engage the patient and family.

The study's primary objective is to evaluate the process, clinical and economic outcomes of telerehabilitation in comparison with usual care. Their secondary objective is to explore and describe contextual factors (both personal and environmental) that will help the delivery of stroke rehabilitation. At the end of the 12-week telerehab intervention, a subset of participants will be purposively selected for an in-depth interview about their experiences with the program.

This team received a CIHR bridge grant in 2016 to test a telerehabilitation platform at two facilities. This application has been developed by an interdisciplinary team of researchers, local healthcare providers from each region involved, two patient partners who have experienced stroke, provincial network stakeholders, and decision makers, all engaged in the successful implementation of stroke guidelines and jurisdictional strategic plans. They also have a panel of health system knowledge users to enhance knowledge translation, starting in Quebec and Alberta. The study has been endorsed by the Quebec Ministry of Health, and the Quebec Heart and Stroke Foundation.

Strengths and Weaknesses/Forces et faiblesses:

Strengths

The proposal is well written with great attention to details. The team has done a stellar job in addressing the previous reviewers' concerns.

This is a strong team with complementary experience. NPA Gaboury was trained in biostatistics, with over 13 years of experience in conducting research with a mixed quantitative-qualitative design. She has published 52 papers in the last 7 years; many of which are related to health service delivery. Co-PI Tousignant held a research chair in telerehabilitation. The team also includes two patient representatives, and a number of experts in stroke rehabilitation and shared decision making.

The conceptual framework on human factors for the telerehab program design is sound. The team takes into consideration the influence of patient impairment, treatment space, training and technology in the intervention design. They pilot study has demonstrated acceptability and feasibility for implementing the program, as well as preliminary efficacy in quality of life.

The study was co-designed with stakeholders, including patients. The primary outcome was identified by patients and health professionals at the pilot as the most meaningful for assessing effectiveness of tele-rehabilitation.

The integrated KT plan is well-articulated. Appropriate knowledge users, including representatives from Quebec Ministry of Health and Alberta Health Services are involved in the study; the collaborations hold great promises for scaling-up in the future.

Weaknesses

The primary outcome is patients' adherence to their stroke rehabilitation plan during the 12 week period. This is operationalized as the time spent doing any stroke rehabilitation exercise. For the intervention period, there is a detailed plan for collecting information about their exercise adherence, such as recording the online sessions, health professional documentations, and journaling by patients. The process for collecting this information is less well defined, however, for patients treated during the control period. Notably, it is expected less than two-thirds of the patients during that period will receive an inter-professional care plan. Since the care during the control period is less cohesive, this may affect patient's self-reporting. Hence, if low exercise participation is found during the control period compared to the intervention period, it would be difficult to discern if it is due to the rehab service delivery, or due to poor reporting by patients during the control period.

Informal caregiver will be recruited to document their experience of care during the intervention period. It is unclear if this will also be done during the control period.

Cost analysis: Since most people recovering from stroke have other health conditions, it might be hard for participants to tease out just the stroke-associated expenses. Also, it would be helpful to clarify whether informal caregiver costs will be

collected (e.g., lost days of work; childcare cost when attending a medical appointment with the patient). Finally how feasible is it to obtain self-reported cost information in the previous 6 months in this population? Recall bias may be an issue. Would it be possible to collect this information monthly?

**Review Type / Type d'évaluation:**	Reviewer 1 / Évaluateur 1
**Name of Applicant / Nom du chercheur:**	Gaboury, Isabelle
**Application No. / Numéro de demande:**	425748
**Agency / Agence:**	CIHR/IRSC
**Competition / Concours:**	Project Grant/Subvention Projet
**Committee / Comité:**	Movement & Exercise/Mouvement et exercice
**Title / Titre:**	Optimizing patient adherence to stroke rehabilitation treatment: a telerehabilitation trial

Budget Recommendation/Recommandation budgétaire:

A budget has been included for expert panel members to attend a workshop at the Canadian Stroke Conference, but no honoraria have been budgeted for patient partners and family caregivers who partake as members in the research team throughout the 5-year study.

Please indicate your appraisal of the integration of sex as a biological variable as a strength, weakness, or not applicable to the proposal./Prière de sélectionner une option pour donner votre évaluation de l'intégration du sexe comme variable biologique en tant que point fort ou point faible de la proposition, ou en tant qu'élément non applicable à la proposition.

- ◉ **Strength/Point fort**
- ◯ **Weakness/Point faible**
- ◯ **Not applicable/Non applicable**

Please indicate your appraisal of the integration of gender as a socio-cultural determinant of health as a strength, weakness, or not applicable to the proposal./Prière de sélectionner une option pour donner votre évaluation de l'intégration du genre comme déterminant socioculturel de la santé en tant que point fort ou point faible de la proposition, ou en tant qu'élément non applicable à la proposition.

- ◉ **Strength/Point fort**
- ◯ **Weakness/Point faible**
- ◯ **Not applicable/Non applicable**

Sex and/or Gender Considerations/Notions de sexe et/ou de genre:

Since the pilot study recruited mostly women, the investigators have explained that they will tailor the recruitment strategy for the RCT to ensure a balance of men and women. Further information about the recruitment strategy would be helpful. Objective 1 will include a subgroup analysis to examine the effect of sex and gender on rehabilitation (exercise) adherence, as well as a number of clinical and process outcomes. There is a brief mention that gender will be taken into consideration in the qualitative analysis in Objective 2, but no detail is provided. It seems that the team may be missing an opportunity to examine contextual factors through a gender lens from the perspectives of health care providers, patients, and their caregivers. However, these issues are addressable.

**Review Type / Type d'évaluation:**	Reviewer 2 / Évaluateur 2
**Name of Applicant / Nom du chercheur:**	Gaboury, Isabelle
**Application No. / Numéro de demande:**	425748
**Agency / Agence:**	CIHR/IRSC
**Competition / Concours:**	Project Grant/Subvention Projet
**Committee / Comité:**	Movement & Exercise/Mouvement et exercice
**Title / Titre:**	Optimizing patient adherence to stroke rehabilitation treatment: a telerehabilitation trial

Adjudication Criteria/Critères de sélection

Significance and Impact of the Research/Importance et impact de la recherche: 4.7

Approaches and Methods/Approches et méthodes: 4.7

Expertise, Experience and Resources/Expertise, expérience et ressources: 4.7

Top/Bottom Selection/Groupe supérieur/inférieur

- (●) **Top/Groupe supérieur**
- ( ) **Bottom/Groupe inférieur**

**Review Type / Type d'évaluation:**	Reviewer 2 / Évaluateur 2
**Name of Applicant / Nom du chercheur:**	Gaboury, Isabelle
**Application No. / Numéro de demande:**	425748
**Agency / Agence:**	CIHR/IRSC
**Competition / Concours:**	Project Grant/Subvention Projet
**Committee / Comité:**	Movement & Exercise/Mouvement et exercice
**Title / Titre:**	Optimizing patient adherence to stroke rehabilitation treatment: a telerehabilitation trial

Summary of Application/Résumé de la demande:

Optimizing patient adherence to stroke rehab treatment: study proposes a telerehab pragmatic clinical trial. 148 patients will receive a remote telemedicine rehab, 148 will be control receiving standard care.
Main objectives are to evaluate the process, clinical and cost outcomes of the interprofessional telerehab intervention and to identify key contextual factors (personal and environmental) related to the outcome of the intervention. 5 sites (rehab centres) will deliver the programme (both control and intervention). Mixed methods study with qualitative interviews to assess the contextual factors of success of delivery of the rehabilitation.
The main outcome is (i) week 12 adherence to the intervention. Secondary outcomes include functional recovery, reintegration to normal living, occurrence of stroke-related complications, adverse events, quality of services. (ii) contextual factors associated with outcomes

**Review Type / Type d'évaluation:**	Reviewer 2 / Évaluateur 2
**Name of Applicant / Nom du chercheur:**	Gaboury, Isabelle
**Application No. / Numéro de demande:**	425748
**Agency / Agence:**	CIHR/IRSC
**Competition / Concours:**	Project Grant/Subvention Projet
**Committee / Comité:**	Movement & Exercise/Mouvement et exercice
**Title / Titre:**	Optimizing patient adherence to stroke rehabilitation treatment: a telerehabilitation trial

Strengths and Weaknesses/Forces et faiblesses:

This pragmatic trial is conducted with knowledge users who are involved throughout providing a compelling iKT approach. Very well written and compelling rationale. The detail of the methodology is excellent and easy to follow. Feasibility is clear and the tele-rehab platform has been utilised successfully in pilot work. The mixed-methods design is appropriate for the outcomes.

The team have done an excellent job of addressing concerns, and the explanation and detail for the self-report adherence, and not using accelerometry is compelling, particularly given how easy it will be to overwhelm the participants. It is also worth noting that accelerometry can be problematic- missing data, use of cut-points etc. The addition of the scale-up plan is commendable. The potential use of the StREAM tool to assess adherence also strengthens to project.
Strong team – PI has more than 13 years using mixed methods/designing intervention and is joined by a multidisciplinary team

**Review Type / Type d'évaluation:**	Reviewer 2 / Évaluateur 2
**Name of Applicant / Nom du chercheur:**	Gaboury, Isabelle
**Application No. / Numéro de demande:**	425748
**Agency / Agence:**	CIHR/IRSC
**Competition / Concours:**	Project Grant/Subvention Projet
**Committee / Comité:**	Movement & Exercise/Mouvement et exercice
**Title / Titre:**	Optimizing patient adherence to stroke rehabilitation treatment: a telerehabilitation trial

Budget Recommendation/Recommandation budgétaire:

Sensible and well justified

Please indicate your appraisal of the integration of sex as a biological variable as a strength, weakness, or not applicable to the proposal./Prière de sélectionner une option pour donner votre évaluation de l'intégration du sexe comme variable biologique en tant que point fort ou point faible de la proposition, ou en tant qu'élément non applicable à la proposition.

- (●) **Strength/Point fort**
- ( ) **Weakness/Point faible**
- ( ) **Not applicable/Non applicable**

Please indicate your appraisal of the integration of gender as a socio-cultural determinant of health as a strength, weakness, or not applicable to the proposal./Prière de sélectionner une option pour donner votre évaluation de l'intégration du genre comme déterminant socioculturel de la santé en tant que point fort ou point faible de la proposition, ou en tant qu'élément non applicable à la proposition.

- (●) **Strength/Point fort**
- ( ) **Weakness/Point faible**
- ( ) **Not applicable/Non applicable**

**Review Type / Type d'évaluation:**	Reviewer 2 / Évaluateur 2
**Name of Applicant / Nom du chercheur:**	Gaboury, Isabelle
**Application No. / Numéro de demande:**	425748
**Agency / Agence:**	CIHR/IRSC
**Competition / Concours:**	Project Grant/Subvention Projet
**Committee / Comité:**	Movement & Exercise/Mouvement et exercice
**Title / Titre:**	Optimizing patient adherence to stroke rehabilitation treatment: a telerehabilitation trial

Sex and/or Gender Considerations/Notions de sexe et/ou de genre:

Subgroup analyses allow for consideration of sex quantitatively and gender from qualitative data.

**Review Type / Type d'évaluation:**	Reviewer 3 / Évaluateur 3
**Name of Applicant / Nom du chercheur:**	Gaboury, Isabelle
**Application No. / Numéro de demande:**	425748
**Agency / Agence:**	CIHR/IRSC
**Competition / Concours:**	Project Grant/Subvention Projet
**Committee / Comité:**	Movement & Exercise/Mouvement et exercice
**Title / Titre:**	Optimizing patient adherence to stroke rehabilitation treatment: a telerehabilitation trial

Adjudication Criteria/Critères de sélection

Significance and Impact of the Research/Importance et impact de la recherche: 4.6

Approaches and Methods/Approches et méthodes: 4.4

Expertise, Experience and Resources/Expertise, expérience et ressources: 4.6

Top/Bottom Selection/Groupe supérieur/inférieur

- (●) **Top/Groupe supérieur**
- ( ) **Bottom/Groupe inférieur**

**Review Type / Type d'évaluation:**	Reviewer 3 / Évaluateur 3
**Name of Applicant / Nom du chercheur:**	Gaboury, Isabelle
**Application No. / Numéro de demande:**	425748
**Agency / Agence:**	CIHR/IRSC
**Competition / Concours:**	Project Grant/Subvention Projet
**Committee / Comité:**	Movement & Exercise/Mouvement et exercice
**Title / Titre:**	Optimizing patient adherence to stroke rehabilitation treatment: a telerehabilitation trial

Summary of Application/Résumé de la demande:

Evidence supports beginning rehabilitation as soon as the patient's medical status has stabilized. Access, adherence, as well as opportunities for structured interprofessional communication among service providers, and failures to engage the patient and family members in a structured decision making process remain challenges.
The aim of this mixed methods pragmatic clinical trial is to evaluate an intervention that provides patients who have experienced stroke the opportunity to return home safely after their acute hospital stay, to encourage patient/family engagement in the rehabilitation, and to overcome challenges of access to patient-centered interprofessional rehabilitation care.

Interrupted time series design of 148 telerehabilitation rehab vs. standard of care. Primary study objective is to evaluate process, clinical outcomes and costs of telerehabilitation. Second objective is to explore and describe contextual factors that will help the delivery of care, and improve patient's outcomes while fully using technology to deliver stroke rehabilitation care.

**Review Type / Type d'évaluation:**	Reviewer 3 / Évaluateur 3
**Name of Applicant / Nom du chercheur:**	Gaboury, Isabelle
**Application No. / Numéro de demande:**	425748
**Agency / Agence:**	CIHR/IRSC
**Competition / Concours:**	Project Grant/Subvention Projet
**Committee / Comité:**	Movement & Exercise/Mouvement et exercice
**Title / Titre:**	Optimizing patient adherence to stroke rehabilitation treatment: a telerehabilitation trial

Strengths and Weaknesses/Forces et faiblesses:

Strengths:

Overall a very well written grant.

Access and adherence in stroke rehab are key. This is a very important, timely project.

Great use of telehealth technology to facilitate timely access to stroke rehab.

Proposal aligns with national guidelines.

Use of rural and urban sites a real strength.

Comprehensive secondary outcomes.

Evaluation of process outcomes a strength.

Integrated Knowledge Transfer approach is a major strength of this project.

Great involvement stakeholders/operational leaders including Quebec Ministry of Health.

Dr. GAboury is a well-trained new investigator who has received a great deal of funding recently. Excellent productivity (52 papers over the past year). Some of the significant contributions was difficult to understand because of my rusty French reading comprehension. Strong team w/ expertise in stroke, KT, telehealth.

Project is well supported from the community, with many letters of support.

Weakness:

Interrupted time series is a logically approach for the study. Would be ideal to ensure sites transition at different times. The plan is to have sites transition once they've reached their target enrollment for controls. The risk is that sites could all transition around the same time/season, and that variance across time/season may be a factor. Seasonal variance is acknowledged, and this is will hopefully be accounted for statistically.

For the control group, will there be tracking of the proportion who receives a) no telerehabilitation, vs. b) interdisciplinary meetings not systematically organized vs. c) interprofessional care plans? This is unclear.

Appreciate the response to reviewers regarding self-reported rehab. Investigators should consider a physical activity monitor as an adjunct to directly record movement. This is especially important in the control arm, as there is no telehealth program, and therefore rehab data will be obtained exclusively through self-report. Objective monitoring was brought up in previous reviews, and the investigators do respond to the criticism. I would suggest that simple technology like FITBIT would require very little effort on the patient, but could confirm movement (and therefore participation) in exercise.

Stroke-related expenses data collection is somewhat vague. Are standard costing models being used to calculate all these? No information/references are given to provide insight as to how these are estimated.

**Review Type / Type d'évaluation:**	Reviewer 3 / Évaluateur 3
**Name of Applicant / Nom du chercheur:**	Gaboury, Isabelle
**Application No. / Numéro de demande:**	425748
**Agency / Agence:**	CIHR/IRSC
**Competition / Concours:**	Project Grant/Subvention Projet
**Committee / Comité:**	Movement & Exercise/Mouvement et exercice
**Title / Titre:**	Optimizing patient adherence to stroke rehabilitation treatment: a telerehabilitation trial

Budget Recommendation/Recommandation budgétaire:

Budget appears appropriate.

Please indicate your appraisal of the integration of sex as a biological variable as a strength, weakness, or not applicable to the proposal./Prière de sélectionner une option pour donner votre évaluation de l'intégration du sexe comme variable biologique en tant que point fort ou point faible de la proposition, ou en tant qu'élément non applicable à la proposition.

- ◉ **Strength/Point fort**
- ◯ **Weakness/Point faible**
- ◯ **Not applicable/Non applicable**

Please indicate your appraisal of the integration of gender as a socio-cultural determinant of health as a strength, weakness, or not applicable to the proposal./Prière de sélectionner une option pour donner votre évaluation de l'intégration du genre comme déterminant socioculturel de la santé en tant que point fort ou point faible de la proposition, ou en tant qu'élément non applicable à la proposition.

- ◉ **Strength/Point fort**
- ◯ **Weakness/Point faible**
- ◯ **Not applicable/Non applicable**

**Review Type / Type d'évaluation:**	Reviewer 3 / Évaluateur 3
**Name of Applicant / Nom du chercheur:**	Gaboury, Isabelle
**Application No. / Numéro de demande:**	425748
**Agency / Agence:**	CIHR/IRSC
**Competition / Concours:**	Project Grant/Subvention Projet
**Committee / Comité:**	Movement & Exercise/Mouvement et exercice
**Title / Titre:**	Optimizing patient adherence to stroke rehabilitation treatment: a telerehabilitation trial

Sex and/or Gender Considerations/Notions de sexe et/ou de genre:

Sub-group analysis will be conducted of sex/gender for both quantitative and qualitative outcomes.

Review Type/Type d'évaluation: SO Notes /Notes de l'agent scientifique
Name of Applicant/Nom du chercheur: Gaboury, Isabelle
Application No./Numéro de demande: 425748
Agency/Agence: CIHR/IRSC
Competition/Concours: 2019-09-11 Project Grant/Subvention Projet
Committee/Comité: Movement & Exercise/Mouvement et exercice
Title/Titre: Optimizing patient adherence to stroke rehabilitation treatment: a telerehabilitation trial

---

Assessment/Évaluation:

Strengths:

Pragmatic clinical trial that involves pre-intervention training on key aspects of the approach and decision making. Involvement of care giver and other knowledge users (especially health authority and government stakeholders) was identified as an important inclusion in the proposal. Clinical and economic outcomes are involved, and personal and environmental contextual factors are nicely included. Pilot data support approach, and barriers faced during the pilot study were addressed in this proposal.

Conceptual framework of the telerehabilitation program are well described. The potential for application of this framework of telerehabilitation to other diseases and models of care was discussed, and seen as an important contribution. Integrated KT plan was well thought out and described.

Sex and gender balance is considered a strength.

Weaknesses:

The definition of patient adherence was well defined and encompasses a number of approaches (e.g. rehabilitation time, ADL usage). While engagement of patients in the experimental arm is expected to be high, it was unclear whether any potential non-adherence that is reported would be related to lack of rehabilitation participation or lack of reporting ability. While this approach may represent standard care, whether this represents a strength or a weakness was discussed. Specifically, while expected in clinical care, the reduced quality of the data may impact the findings in a pragmatic trial. Implementation of strategies to mitigate this issue and improve the quality of the findings are suggested.

Given that the cost analysis is over a 6-month period, lack of recall from participants may reduce the fidelity of the data. Suggest reducing the timeframe to 3 months maximum.

Budget:

No concerns with budget or term.

****

Note: The final rating of the application, provided in the Notice of Decision (NOD), is the averaged rating of the peer review committee members following the discussion of the application during the committee meeting, and therefore may differ from the ratings provided by the assigned reviewers in their respective reviews.

Review Type/Type d'évaluation: SO Notes /Notes de l'agent scientifique
Name of Applicant/Nom du chercheur: Gaboury, Isabelle
Application No./Numéro de demande: 425748
Agency/Agence: CIHR/IRSC
Competition/Concours: 2019-09-11 Project Grant/Subvention Projet
Committee/Comité: Movement & Exercise/Mouvement et exercice
Title/Titre: Optimizing patient adherence to stroke rehabilitation treatment: a telerehabilitation trial

---

Assessment/Évaluation:

Remarque : La cote définitive de la demande, qui apparaît dans l'avis de décision, représente la moyenne des cotes accordées par les membres du comité d' évaluation par les pairs après avoir débattu de la demande à la réunion du comité. Elle peut donc différer de celle donnée par les évaluateurs dans leur évaluation respective.

````````````````````````````````````````````````````````````````````````````````````````````````````````````````````````````````````
````````````````````````````````````````````````````````````````````````````````````````````````````````````````````````````````````
